# Immune-complex glomerulonephritis with a membranoproliferative pattern in Frasier syndrome: a case report and review of the literature

**DOI:** 10.1186/s12882-020-02007-0

**Published:** 2020-08-24

**Authors:** Daisuke Matsuoka, Shunsuke Noda, Motoko Kamiya, Yoshihiko Hidaka, Hisashi Shimojo, Yasushi Yamada, Tsutomu Miyamoto, Kandai Nozu, Kazumoto Iijima, Hiroyasu Tsukaguchi

**Affiliations:** 1 Department of Pediatrics, Shinshu University School of Medicine, Matsumoto, Japan; 2 Department of Pediatrics, Nagano Red Cross Hospital, Nagano, Japan; 3 Center for Medical Genetics, Shinshu University Hospital, Matsumoto, Japan; 4 Department of Pathology, Aizawa Hospital, Matsumoto, Japan; 5 Department of Obstetrics and Gynecology, Shinshu University School of Medicine, Matsumoto, Japan; 6 Department of Pediatrics, Kobe University Graduate School of Medicine, Kobe, Japan; 7 Second Department of Internal Medicine, Division of Nephrology, Kansai Medical University, 2-5-1 Shinmachi Hirakata, Osaka, 573-1191 Japan

**Keywords:** Focal segmental glomerulosclerosis, Frasier syndrome, Membranoproliferative glomerulonephritis, Wilms tumor

## Abstract

**Background:**

Mutations in the *Wilms tumor 1* gene cause a spectrum of podocytopathy ranging from diffuse mesangial sclerosis to focal segmental glomerulosclerosis. In a considerable fraction of patients with *Wilms tumor 1* mutations, the distinctive histology of immune-complex-type glomerulonephritis has been reported. However, the clinical relevance and etiologic mechanisms remain unknown.

**Case presentation:**

A 5-year-old child presented with steroid-resistant nephrotic range proteinuria. Initial renal biopsy revealed predominant diffuse mesangial proliferation with a double-contour and coexisting milder changes of focal segmental glomerulosclerosis. Immunofluorescence and electron microscopy revealed a full-house-pattern deposition of immune complexes in the subendothelial and paramesangial areas. Serial biopsies at 6 and 8 years of age revealed that more remarkable changes of focal segmental glomerulosclerosis had developed on top of the initial proliferative glomerulonephritis. Identification of a de novo *Wilms tumor 1* splice donor-site mutation in intron 9 (NM_024426.6:c.1447 + 4C > T) and 46,XY-gonadal dysgenesis led to the diagnosis of Frasier syndrome.

**Conclusions:**

Our findings, together with those of others, point to the importance of heterogeneity in clinicopathological phenotypes caused by *Wilms tumor 1* mutations and suggest that immune-complex-mediated membranoproliferative glomerulopathy should be considered as a histological variant.

## Background

Mutations in *Wilms tumor 1* (*WT1*) gene cause several diseases characterized by renal and /or genital anomalies, such as Denys–Drash syndrome (DDS), Frasier syndrome (FS), and isolated focal segmental glomerulosclerosis (FSGS). DDS patients typically present early-onset diffuse mesangial sclerosis (DMS), a 46,XY disorder of sex differentiation, and Wilms tumor (WT). FS patients tend to exhibit milder phenotypes with an onset at adolescence, including FSGS, male-to-female sex reversal, and gonadoblastoma, but usually lack WT [1]. Given the high incidence of WT and gonadoblastoma in DDS and FS, prophylactic gonadectomy and nephrectomies are recommended [1]. Over 95% of DDS patients carry missense mutations in exons 8 and 9, whereas FS is commonly caused by a splice-donor site mutation in intron 9 [1]. *WT1*-related nephropathy is generally ascribed to developmental defects in glomerular podocytes [2, 3]. Several patients with DDS or FS display membranoproliferative glomerulonephritis (MPGN) that is mainly characterized by subendothelial immune deposits [3–9], suggesting that renal pathologies resulting from *WT1* mutations are complex and affected by multiple factors. Possible pathogenic mechanisms are discussed by reviewing the current literature. Here, we present a case of a child with FS. To the best of our knowledge, this is the first case report where renal histological changes have been followed up for 4 years before and after immunosuppressive therapy. Our case report should alert clinicians to consider the possible existence or *WT1* mutations behind seemingly immunologic forms of MPGN.

## Case presentation

A child was first diagnosed with proteinuria during a regular check-up at 3 years of age but remained untreated until the age of 5 years old, when proteinuria reached the nephrotic range. Since the proteinuria did not resolve after 4 weeks of oral prednisolone (2 mg/kg/day), the child was referred to our hospital for further evaluation.

**Fig. 1:**
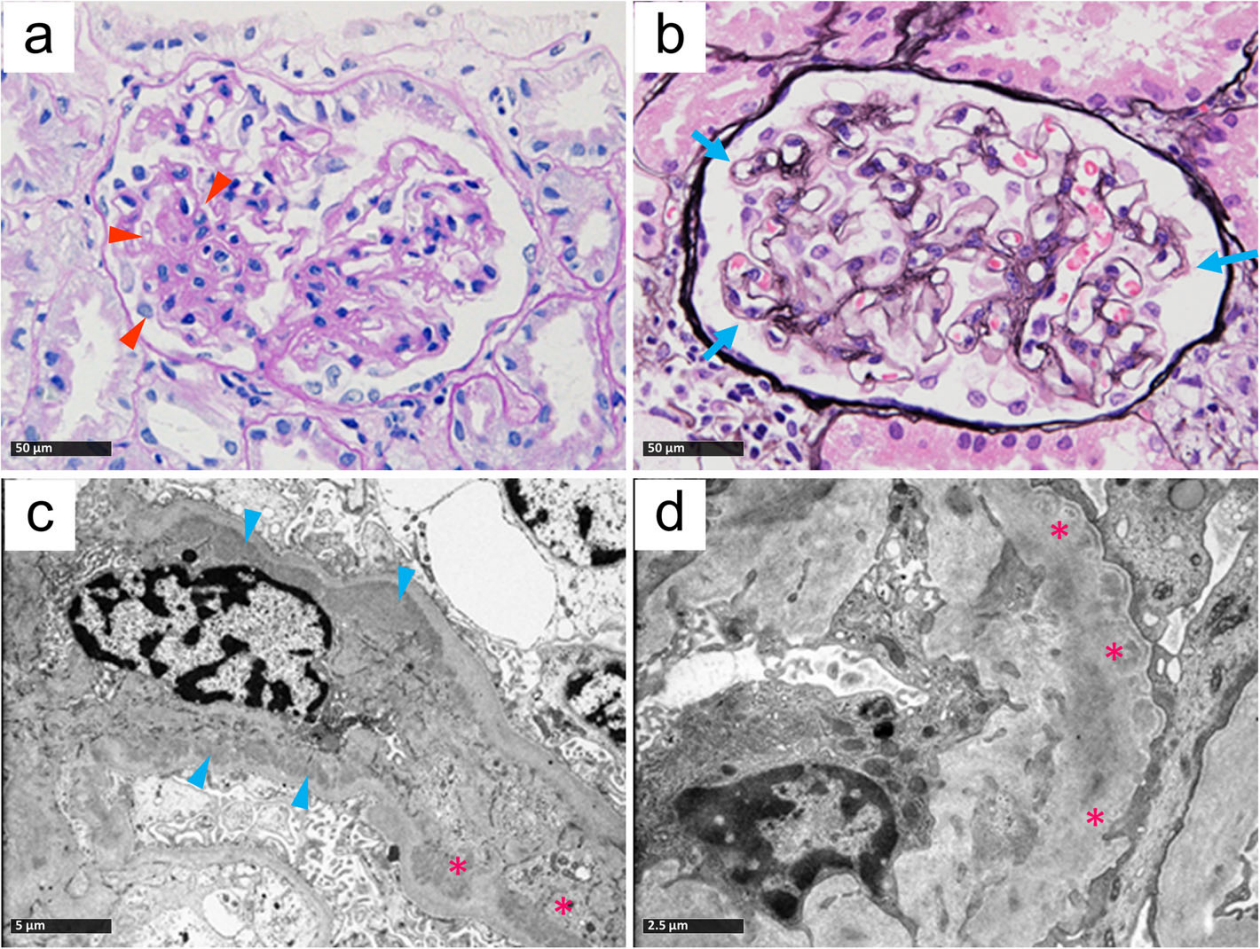
Representative light and electron microscopy images of the first and second biopsies. **a** Mesangial proliferation with segmental sclerosis (arrowheads; periodic acid-Schiff staining; original magnification, 400×). **b** Double-contour formation (arrows; periodic acid methenamine silver staining; original magnification, 400×) (**c**) Subendothelial (asterisks) and paramesangial (arrowheads) deposits (electron micrograph; original magnification, 4000×). (a–c) Images from the first biopsy at age 5. **d** Irregularities of the GBM (asterisks) with mesangial interposition (asterisks; electron micrograph; original magnification, 8000×). **d** Image from the second biopsy at age 6. GBM: glomerular basement membrane

Pregnancy and perinatal periods were uneventful, with no family history of kidney disease. Physical examination revealed no edema, rash, or arthralgia, and the child had normal female external genitalia. Laboratory studies indicated nephrotic syndrome: hypoalbuminemia (2.3 g/dL), hyperlipidemia (total cholesterol, 342 mg/dL), and massive proteinuria (11 g/g creatinine) without hematuria. Blood-cell count, renal function, and serum complement C3 and C4 levels were all normal. Serological tests for hepatitis B and C and anti-nuclear antibodies were negative. In the first renal biopsy at age 5 (See Additional files 1, 2, 3 and 4), ~ 50% of glomeruli displayed global mild-to-severe mesangial proliferation, whereas others showed FSGS (Fig. 1a). In some glomerular tufts, the capillary wall was irregularly thickened with a double-contour configuration (Fig. 1b). Foam cells had infiltrated focally around the tubular interstitium. Immunofluorescence revealed a coarsely granular, full-house deposition pattern of 1+, positive for IgG, IgM, IgA, C3, and C1q along with trace C4+/− labeling in the mesangial and peripheral capillary loops. Electron microscopy revealed electron-dense deposits in the subendothelial and paramesangial areas (Fig. 1c). The glomerular basement membranes (GBM) exhibited normal thickness and contour, while focally showing subendothelial widening. These histologic features were consistent with endocapillary immune-complex (IC) glomerulonephritis with partial MPGN pattern, whereas minor FSGS changes were occasionally observed. Based on pathological assessment, intravenous methylprednisolone pulse, intravenous cyclophosphamide, cyclosporine A, and mycophenolate mofetil were administered. However, the proteinuria was unresponsive.

The second biopsy at age 6 (See Additional files 5, 6 and 7), following intravenous methylprednisolone pulse and cyclophosphamide, showed remarkable attenuation of IgA, C3, and C1q depositions in mesangiocapillary areas relative to the first biopsy. Irregular GBM thickening was more apparent with a double-contour pattern due to mesangial interposition (Fig. 1d). Along with a stepwise increase in cyclosporine A dosage, proteinuria gradually declined (3 g/g creatinine), thereby achieving partial remission. The third biopsy at age 8 (See Additional files 8 and 9) revealed coarse granular deposits of IgM (2+) and C3(1+) at the capillary periphery, suggesting macromolecule entrapment in sclerosing glomeruli, and we observed significantly fewer foam cells around the tubular interstitium. Throughout the clinical course, IC depositions had ameliorated in response to immunosuppressive therapy. However, as the child aged, glomerular capillary remodeling and podocyte injuries had progressed, thereby causing the development of more remarkable FSGS features onto the initial proliferative glomerulopathy.

**Fig. 2:**
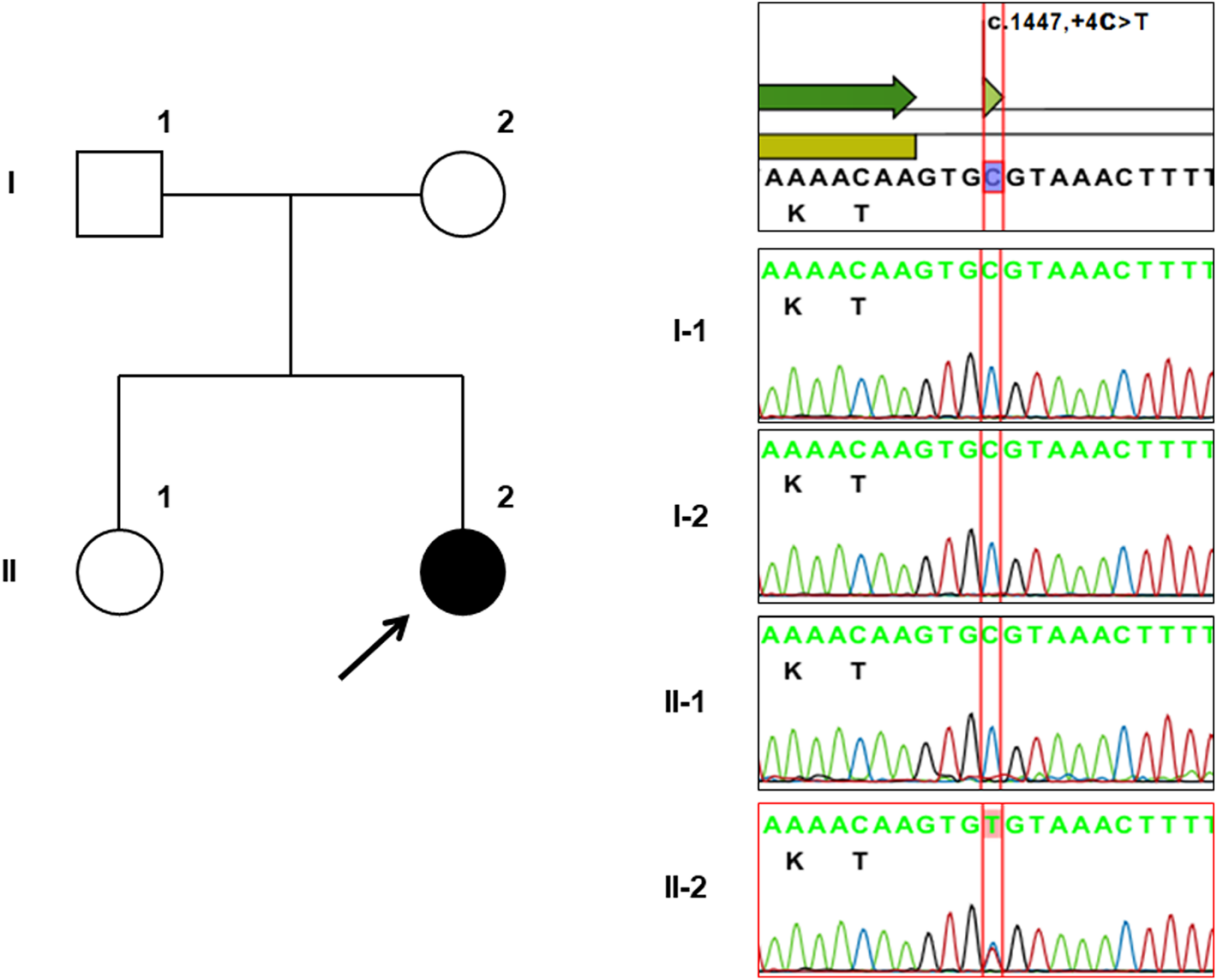
Pedigree and sequencing analyses. Sanger sequencing of the *WT1* exon 9 and intron 9 boundary in the affected individual and family members. The affected child (II-1, proband shown by arrow) harbored a single nucleotide substitution in the canonic donor KTS splice site of *WT1* intron 9 (IVS9; c.1447 + 4C > T; RefSeq NM_024426.6:WT1 isoform D; ClinVar:000003500, dbSNP:rs587776577), which was absent in family members, indicating a de novo mutation. This variant has been reported elsewhere under alternate variant designations (e.g., 1432 + 4C > T or IVS9 + 4C > T). *WT1*: Wilms tumor suppressor gene

Because of the steroid-resistant nephrotic syndrome, genetic testing was recommended. Targeted sequencing for 88 known renal disease genes (Additional files 10) detected a splice-donor site mutation in *WT1* intron 9 (NM_024426.6: c.1447 + 4C > T). Segregation analysis of family members confirmed a de novo mutation (Fig. 2). Subsequent G-band analysis revealed a 46,XY karyotype. Bilateral streak gonads were observed by magnetic resonance imaging, confirming FS diagnosis. The patient underwent gonadectomy at age 10, was diagnosed with gonadoblastoma, and is currently treated with cyclosporine A and an angiotensin II-receptor blocker. The proteinuria is now in the nephrotic range, but renal function remains normal.

## Discussion and conclusions

Herein, we presented a case of a child with FS with steroid-resistant nephrotic syndrome, whose renal histology initially showed a predominant proliferative glomerulonephritis that later progressed into FSGS. The first renal histology at age 5 was consistent with mesangial proliferative glomerulonephritis with MPGN pattern diagnosis based on mesangial proliferation, double-contour GBM, and “full-house” granular IC deposits along the glomerular capillary loops, as well as the paramesangial region. The pathologic findings were indistinguishable from those commonly seen in lupus nephritis, although our patient lacked serological abnormalities. The second and third biopsies, following immunosuppressive therapy, at ages 6 and 8, respectively, revealed that FSGS features (i.e., focal segmental capillary obsolescence and tubule interstitial foam cell infiltration) were superimposed on the IC glomerulonephritis and had become more apparent. Despite a partial response, persistent steroid-resistant nephrotic proteinuria incentivized us to conduct genetic testing, thereby allowing the diagnosis of FS caused by a typical splice-donor mutation in intron 9 of *WT1*.

*WT1* mutations can cause a broad spectrum of clinical diseases affecting urogenital development and sexual differentiation at variable severity and combinations. Mutational survey of *WT1* in steroid-resistant nephrotic syndrome cohorts [2, 3] revealed that an intron 9 splice mutation typically causes FSGS with a gonadal tumor, whereas missense and truncating mutations result in DMS with nephroblastoma. However, morphologic abnormalities considerably vary in histologic appearance among individuals with DDS/FS [3–9]. Detailed analysis of the renal histology of DDS individuals revealed complex glomerular changes, including endotheliosis-like endothelial injuries, foot-process fusion, and GBM alterations [6]. Previous studies report a significant fraction of DDS/FS individuals, including original and some familial cases, display MPGN with IC deposition in addition to FSGS or DMS, a histopathology commonly seen in *WT1*-related glomerulopathy [3–17] (Tables 1 and 2). Out of six DDS cases, four patients harbored a p.Arg467Trp (NM_024426.6:c.1399C > T) variant [4–6], the most common substitution (present in 40% of DDS patients), and two harbored a nonsense p.Arg463Ter (NM_024426.6:c.1387C > T) variant [3, 7] manifesting in an MPGN pattern. Moreover, nine FS cases have been reported, including two monozygous twins harboring a donor splice site mutation in intron 9 and initially presenting with MPGN [8, 9]. In these cases, ICs, comprised of either full-house or combined IgG + C3 patterns, were deposited along glomerular capillaries. Glomerulopathy in DDS usually manifests earlier and progresses faster into end-stage renal disease, relative to FS. Notably, there are DDS cases initially presenting with thrombotic microangiopathy or atypical hemolytic uremia syndrome (HUS) [18–20] (Table 3). It is not clear how the *WT 1* variant could facilitate HUS-like severe endothelial injuries. Co-occurrence of atypical HUS with other glomerular diseases (e.g. FSGS) has been reported [21], suggesting that complement activation and podocyte dysfunction may be related, mechanistically. However, to address this hypothesis, we need the description of further case reports as well as additional data collection from experimental studies. As Noris et al. suggested [21], we should also take into account the possibility that another genetic abnormality or triggering environmental factor may be the main MPGN etiology in this case, over the *WT1* glomerulopathy background.

**Table 1:** Summary of WT1-related glomerulopathy with MPGN pattern

Reported cases Literature	Clinical diagnosis	Phenotype /Karyotype	*WT1* mutation	NS onset (yr)	ESRD (yr)	Renal Histology				Tumor onset	Surgical resection
						Initial diagnosis (age)	IF	Follow-up (age)	Other features		
*WT1 missense variant*											
Neuhaus TJ (2011) [4]	DDS	Male, 46XY	p.Arg467Trp	4	5	MPGN+DMS (4 yr)	Full-house	DMS (9,12 yr)	Posttransplant recurrence at age 9	WT (1.8 yr)	1.8 yr (L), 5 yr (R)
Karmila AB (2019) [5]	DDS	Male, 46XY	p.Arg467Trp	4	4	MPGN+DMS (4 yr)	IgG, C3		Graft loss at age 12	ND	NA
			p.Arg467Trp	2.5	3	MPGN+FSGS (4 yr)	IgG, C3		No posttransplant recurrence	ND	NA
Schmacher VA (2007) [6]	DDS		p.Arg467Trp	ND		MPGN+DMS	ND		Fibrillar deposits in GBM	ND	1.7 yr
*WT1 nonsense variant*											
Bockenhauer D (2009) [7]	DDS	Female, 46XX	p.Arg463Ter	4	9	MPGN (4 yr)	Full-house		No posttransplant recurrence	WT (1 yr)	1 yr (R), 9 yr (L)
Chernin G (2010) [3]		Female, 46XX	p.Arg463Ter	3.9		MPGN	ND			WT (1 yr)	ND
*WT1 splice variant*											
Ito S (2003) [8]	FS	Female, 46XY^a^	c.1447+4C>T	10		MPGN (13 yr)	Trace		Thin GBM, interstitial foam cells	SG	ND
		Female, 46XY^a^	c.1447+4C>T	10	20	MPGN (10 yr)	C3, IgA		Thin GBM, interstitial foam cells	SG	ND
		Female, 46XY	c.1447+2T>C	10	23	MPGN (11 yr)	IgM, IgG, IgA	FSGS (14 yr)	Thin GBM, interstitial foam cells	GB	19 yr
Aucella F (2006) [9]	FS	Female, 46XY	c.1447+5G>A	5	14	MPGN (6 yr)	IgG, C3	Diffuse GS (10 yr)	Interstitial foam cells	SG	ND
	FS	Female, 46XX	c.1447+5G>A	6	10	MPGN (8 yr)	Absent		Interstitial foam cells	ND	ND
Klamt B (1998) [10]	FS	Female, 46XY	c.1447+5G>A	17	19	FSGS (10 yr)				ND	ND
		Female, 46XX	c.1447+5G>A	9	11	MPGN	IgG, C3			ND	ND
Frasier SD (1970) [11]	FS	Female, 46XY^a^	c.1447+5G>A	3.5	7.5	MPGN			Interstitial foam cells	GB, Te	ND
	FS	Female, 46XY^a^	c.1447+5G>A	3.5		MPGN			Interstitial foam cells	GB	6 yr
Present case	FS	Female, 46XY	c.1447+4C>T	5	NA	MPGN (5 yr)	Full-house	FSGS (8 yr)		GB	11 yr

*WT1* Wilms tumor suppressor gene, *MPGN* membranoproliferative glomerulonephritis, *NS* nephrotic syndrome, *ESRD* end-stage renal disease, *DDS* Denys-Drash syndrome, *DMS* diffuse mesangial sclerosis, *WT* Wilms tumor, *L* left, *R* right, *ND* not determined, *NA* not applicable, *FSGS* focal segmental glomerulosclerosis, *GBM* glomerular basement membrane, *FS* Frasier syndrome, *SG* Streak gonads, *GB* gonadoblastoma, *Te* teratoma

^a^Monozygous twin

The *WT1* variants are shown based on the reference sequence of the longest isoform D trasncript of *WT1* (NM_024426.6) and the recommendation of variant nomenclature from the Human Genome Variation Society. The p.Arg467Trp(c.1399C>T) and p.Arg463Ter(c.1387C>T) variants were originally reported as p.Arg394Trp and p.Arg390X, respectively. The sequence variant nomenclatures of splice variants of c.1447+4C>T, c.1447+2T>C, and c.1447+5G>A are used for those originally reported as IVS9+4C>T, IVS9+2T>C, and IVS9+5G>A, respectively

**Table 2:** Summary of renal manifestations in Denys-Drash syndrome with Wilms tumor

Literature	Karyotype	Phenotype	WT onset (m)	NS or Proteinuria onset (m)	Renal histology	Tumor therapy	Renal outcomes after surgical excision
Drash A (1970) [12]	ND	Ambiguous genitalia	21	12	DMS or MPGN^a^	Right nephrectomy	ESRD at age 7.5 yr
	46XY	Incomplete female genitalia		2	MPGN		ESRD at age 3.4 yr, incidental WT on autopsy
Spear GS (1971) [13]	46XY	Ambiguous genitalia	9	4	MPGN, IgG + C3		incidental WT on autopsy at age 4 m
Barratt TM (1978) [14]	46XY	Ambiguous genitalia	70	39	MPGN, Full-house	Right nephrectomy	
McCoy FE (1983) [15]	46 XY	Ambiguous genitalia	35	35	MPGN	Right nephrectomy + V, A	ESRD
Thorner P (1984) [16]	ND	Female	22	22	MPGN	Right total & left partial nephrectomy	Proteinuria persisted Circulating IC vanished after nephrectomy
Scully RE (1998) [17]	46 XY^b^	Aniridia	17	156	FSGS	V, A + radiation	Proteinuria persisted

*WT* Wilms tumor, *NS* nephrotic syndrome, *ND* not determined, *DMS* diffuse mesangial sclerosis, *MPGN* membranoproliferative glomerulonephritis, *ESRD* end-stage renal disease, *V* Vincristine, *A* actinomycin, *IC* immune complex, *FSGS* focal segmental glomerulosclerosis

^a^ Histology for this patient (case 1) was re-evaluated by Spear GS et al.; *J Pediatr* 1971

^b^ Suspected 11p13 deletion

*WT* mutations were not defined in the listed cases

**Table 3:** Summary of WT1-related glomerulopathy presenting an atypical hemolytic uremic syndrome or thrombotic microangiopathy phenotype

Literature	Clinical diagnosis	Phenotype /Karyotype	*WT1* mutation	NS onset (m)	ESRD (age)	Renal histology			Tumor features
						Initial diagnosis (age)	Follow-up	Other features	
Alge JL (2017) [18]	DDS	Female, 46XX	p.Arg467Trp	8	NA	aHUS		CFH H3 allele	
Sherbotie JR (2000) [19]	DDS	Male, 46XY	p.Thr475fs	13	2.5 yr	aHUS	no recurrence after Tx		Intralobar nephrogenic rest
	DDS	Male, 46XY	p.Arg467Trp	16	5 yr	aHUS	no recurrence after Tx	HT	
Manivel JC (1987) [20]	DDS	Male, 46XY	ND	26	32 m	MPGN + chronic HUS		GB, HT	Nephrectomy and gonadectomy at age 32 m

*WT1* Wilms tumor suppressor gene, *NS* nephrotic syndrome, *ESRD* end-stage renal disease, *DDS* Denys-Drash syndrome, *NA* not applicable, *aHUS* atypical hemolytic uremic syndrome, *CFH* complement factor H, *Tx* treatment, *HT* hypertension, *ND* not determined, *MPGN* membranoproliferative glomerulonephritis, *HUS* hemolytic uremic syndrome, *GB* gonadoblastoma.

The *WT1* variants are shown based on the reference sequence of the longest isoform D transcript of *WT1* (NM_024426.6) and the recommendation of variant nomenclature from the Human Genome Variation Society. The p.Arg467Trp variant was originally reported p.Arg394Trp and other alternate variant designations (e.q., c.1384C>T, 1180C>T, p.Arg462Trp). The p.Thr475fs was originally reported as p.Thr402fs

The mechanisms by which *WT1* mutations cause MPGN have not been defined. Three factors might be implicated in the pathogenesis of IC-mediated glomerulonephritis involving *WT1* mutations: 1) the WT-derived precipitating antigen promoting IC formation, 2) altered immune responses, and 3) increased vulnerability to endothelial injuries in structurally maldeveloped glomerular capillaries. First, MPGN is occasionally associated with malignancy, typically in lymphoproliferative disorders, but also in solid tumors (i.e., lung, colon, and renal carcinoma) [22, 23]. In this context, it is plausible that ICs might form through aberrant immune responses against oncofetal and/or non-autologous tumor antigens and trigger endothelial injuries of glomerular capillaries in WT patients [23]. Clinically, in most DDS cases, glomerulonephritis precedes or manifests simultaneously with WT diagnosis [3–7, 12–16] (Tables 1 and 2). Moreover, nephrotic syndrome can persist, even after complete excision of tumors with no evidence of recurrence and metastasis [4, 7]. However, the existence of WT-specific circulating antibodies has not been well defined. These observations indicate no convincing biological evidence linking WT to MPGN, thereby warranting further study.

Second, dysfunctional *WT1* might be associated with aberrant immune responses, leading to IC formation [22], based on its role in the transcriptional regulation of multiple genes implicated in the differentiation of hematopoietic stem cells and apoptosis [24]. Consistent with our case, clinical studies report the effectiveness of cyclosporin in *WT1* glomerulopathy [25], with no report of MPGN recurrence after renal transplant in patients bearing *WT1* mutations, except for one DDS patient [4], which represents an unusual case of MPGN recurrence in the allografted kidney, even after WT resection and subsequent thorough immunosuppressive therapy.

An MPGN pattern resembling a glomerular morphology distinctive from classic podocytopathy (FSGS/DMS) is recognizable in some DDS/FSGS cases by the disrupted glomerular capillary integrity and, as of yet, undetermined predisposing factors for IC deposition. In-depth evaluation of glomerular histology in DDS patients and mouse models harboring p.Arg467Trp (NM_024426.6:c.1399C > T) demonstrates complex disturbances in podocytes and endothelial cells, as well as GBM maturation [6, 26]. Several studies of FS patients suggest GBM alterations as the first histological changes that precede overt features of MPGN (IC deposits) and FSGS (interstitial foam cells) [8, 9].

This case adds to the evidence of endothelial injuries as essential components in the pathogenesis of *WT1*-related glomerulopathy. In addition to DMS or FSGS pathologies, IC-mediated MPGN should be considered as a histological variant in patients harboring *WT1* mutations. Early recognition of *WT1* mutations allows for personalized choices of immunosuppressive reagents and prevention of tumorigenesis.

## Supplementary information

**Additional file 1: Fig. S1.** Renal histology of the first biopsy at age 5. (a, b) Representative images of the first renal biopsy at age 5. Most glomeruli appear grossly normal. (b) Arrows indicate foam cells in the interstitium. Scale bar, 250 μm. (c) Higher magnification (b) showing foam cell aggregation with a striped appearance (arrowheads) and segmental sclerosis (arrow). There were no inflammatory or sclerotic lesions in the interlobular artery (asterisk). Scale bar, 100 . (d, e) Representative images of glomeruli show mild mesangial proliferation with GBM thickening (arrows) and (d) foam cells in the periglomerular tubular interstitium (arrowhead). (e) Some glomeruli showed tuft adhesion and segmental sclerosis (arrows). Scale bar, 50 μm; periodic acid-Schiff staining.

**Additional file 2: Fig. S2.** Immunofluorescence images of the first renal biopsy at age 5. Immunofluorescence images of renal biopsy at age 5. Immunoglobulins (IgG, IgM, and IgA) and complement proteins (C3 and C1q) were diffusely deposited along the capillary wall and expressed at similar levels.

**Additional file 3: Fig. S3.** Electron micrographs of the first biopsy at age 5. (a) Representative images of electron-dense deposits in the paramesangial area (asterisks). Thickness and contour of the GBM appear generally normal, while there was partial scalloping in the paramesangial region. Scale bar, 2 μm. (b) Enlarged view of the boxed area in (a). Some portion of the GBM was slightly thickened showing double layers of dense matrix (arrowheads). Scale bar, 1 μm. (c) Electron-dense deposits in the subendothelial and paramesangial spaces (asterisks), with occasional thickening of the adjacent GBM. In podocytes, there were numerous cytoplasmic vacuoles and deformities, including foot-process effacement and microvilli formation. Scale bar, 2 μm. (d) Enlarged view of the boxed area in (c). The GBM appeared abnormally thickened with granular, subendothelial, electron-dense deposits (asterisks). Scale bar, 1 μm.

**Additional file 4: Table S1.** Summary of serial immunofluorescence studies.

**Additional file 5: Fig. S4.** Immunofluorescence images of the second renal biopsy at age 6. Representative immunofluorescence images of the second renal biopsy at age 6. Dense IgM deposition (1+) localized in the mesangial area, as well as in the periphery of glomerular capillaries, forming a fringe-like pattern (arrowheads). IgG co-deposited (1+) to a lesser degree than IgA, C3(+/−). Overall, immunocomplex deposition was significantly lower than that found in the first biopsy, indicating successful removal of ICs by immunosuppressive therapy.

**Additional file 6: Fig. S5.** Histology of the second renal biopsy at age 6. (a, b) Representative images of the second renal biopsy at age 6. Most glomeruli showed an increase in mesangial matrices, whereas some displayed segmental sclerosis (arrows). Foam cells accumulated primarily in the interstitium (arrowheads). (a) Scale bar, 250 μm. (b) Scale bar, 100 μm. (c) Increases in mesangial matrix were more pronounced in perihilar regions (arrow). Tubular atrophy and dilatation (asterisk) were observed in the interstitium adjacent to the sclerosing glomeruli. Histology resembled FSGS more closely, despite the coexistence of some MPGN characteristics. Scale bar, 50 μm. (d) Representative image of glomeruli with global mesangial proliferation and aggregation of foam cells within the capillary lumen and Bowman’s space (arrowheads). Some glomeruli exhibited tuft adhesion and segmental sclerosis (arrow). Scale bar, 50 μm; periodic acid-Schiff staining. (a–c) Periodic acid methenamine silver staining.

**Additional file 7: Fig. S6.** Electron micrographs of the second biopsy at age 6. Ultrastructure of glomeruli after immunosuppressive therapy. (a) The amount of capillary deposition decreased relative to that of the first biopsy. However, some deposits remained in the subendothelial (arrow) and subepithelial regions (arrowheads). Double arrows indicate deposit-free capillary wall. Scale bar, 2 μm. (b) Scalloping and irregular thickening of the GBM observed along with electron-lucent matrix expansion (asterisks). Podocytes were deformed with cytoplasmic vacuolization, foot-process effacement, and microvilli formation. Scale bar, 2 μm. (c) Mesangial matrices and fragmented electron-dense depositions (asterisks) increased in the paramesangial regions. Scale bar, 2 μm. (d) The GBM was abnormally thickened and partially split due to an accumulation of fine granular deposits in the subendothelial and subepithelial regions, as well as mesangial interposition. Scale bar, 2 μm.

**Additional file 8: Fig. S7.** Immunofluorescence images of the third renal biopsy at age 8. Dense and diffuse IgM deposition observed predominantly along the glomerular capillary. Complement proteins (C3, C1q, and C4) co-deposited with IgM in the tufts, suggesting non-specific entrapping of macromolecules due to a gradual loss of glomerular structural integrity.

**Additional file 9: Fig. S8.** Renal histology of the third biopsy at age 8. Representative images of the third renal biopsy at age 8. An increasing fraction of glomeruli showed segmental-to-global sclerosis (arrows) and expansion of interstitial fibrosis (asterisks), suggesting FSGS progression Foam cells focally aggregated in the interstitium (arrowheads). However, overall cell density was significantly lower than in previous biopsies. (a) Scale bar, 250 μm. (b) Scale bar, 100 μm. (c) Arrows indicate the double-contour in the glomerular capillary, and the arrowhead indicates segmental luminal dilation and foam cell accumulation. Scale bar, 50 μm. (d, e) Glomerulus with increased mesangial matrix and tuft adhesion (arrow) along with foam cell accumulation in Bowman’s space and the capillary lumen (arrowheads). (e) Double arrowheads indicate hyaline nodules in the vascular pole. Scale bar, 50 μm; The arteriole (asterisk) appeared normal. (a–c) Periodic acid methenamine silver staining, (d, e) periodic acid-Schiff staining.

**Additional file 10: Table S2.** A list of the genes included in the next-generation sequencing panel screening.

**Additional file 11.** CARE Checklist.

## Data Availability

All data generated or analyzed during this study are included in this published article and its additional files.

## Abbreviations

DDS: Denys–Drash syndrome
FS: Frasier syndrome
FSGS: Focal segmental glomerulosclerosis
GBM: Glomerular basement membrane
IC: Immune-complex
MPGN: Membranoproliferative glomerulonephritis
WT: Wilms tumor
*WT1*: *Wilms tumor 1*
