# Supplementary material for: Immune-complex glomerulonephritis with a membranoproliferative pattern in Frasier syndrome: a case report and review of the literature
Source: BMC Nephrol. 2020 Aug 24;21:362. doi: 10.1186/s12882-020-02007-0 (PMC7446187; doi:10.1186/s12882-020-02007-0)
Supplement: Supplementary file 1 — Additional file 1: Fig. S1. Renal histology of the first biopsy at age 5. (a, b) Representative images of the first renal biopsy at age 5. Most glomeruli appear grossly normal. (b) Arrows indicate foam cells in the interstitium. Scale bar, 250 μm. (c) Higher magnification (b) showing foam cell aggregation with a striped appearance (arrowheads) and segmental sclerosis (arrow). There were no inflammatory or sclerotic lesions in the interlobular artery (asterisk). Scale bar, 100 . (d, e) Representative images of glomeruli show mild mesangial proliferation with GBM thickening (arrows) and (d) foam cells in the periglomerular tubular interstitium (arrowhead). (e) Some glomeruli showed tuft adhesion and segmental sclerosis (arrows). Scale bar, 50 μm; periodic acid-Schiff staining. [file 12882_2020_2007_MOESM1_ESM.pdf]

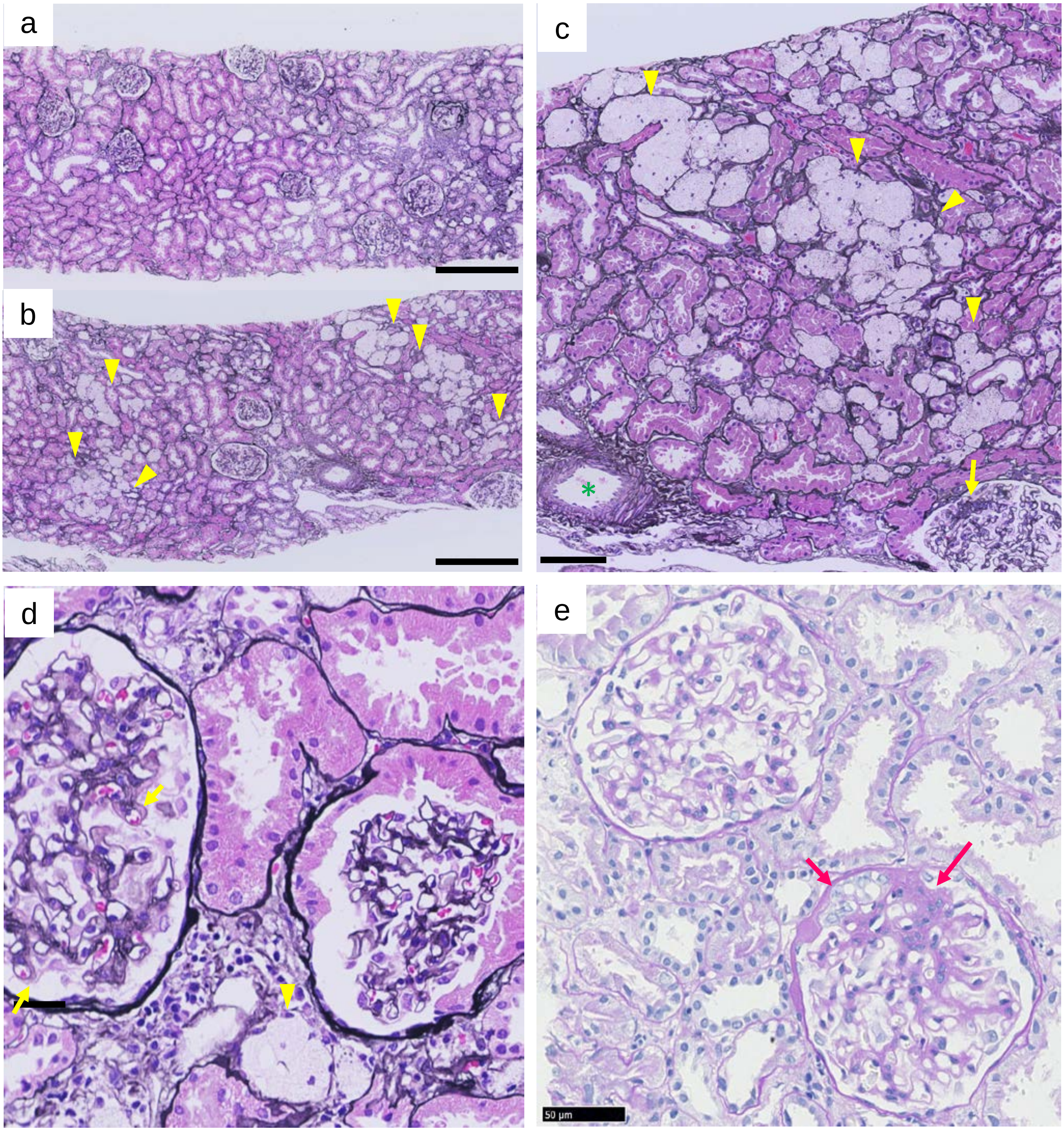

Figure S1. Renal histology of the first biopsy at age 5

(a, b) Representative images of the first renal biopsy at age 5. Most glomeruli appear grossly normal. (b) Arrows indicate foam cells in the interstitium. Scale bar, 250 µm. (c) Higher magnification (b) showing foam cell aggregation with a striped appearance (arrowheads) and segmental sclerosis (arrow). There were no inflammatory or sclerotic lesions in the interlobular artery (asterisk). Scale bar, 100 µm. (d, e) Representative images of glomeruli show mild mesangial proliferation with GBM thickening (arrows) and (d) foam cells in the periglomerular tubular interstitium (arrowhead). (e) Some glomeruli showed tuft adhesion and segmental sclerosis (arrows). Scale bar, 50 µm; periodic acid-Schiff staining.
