# Supplementary material for: Immune-complex glomerulonephritis with a membranoproliferative pattern in Frasier syndrome: a case report and review of the literature
Source: BMC Nephrol. 2020 Aug 24;21:362. doi: 10.1186/s12882-020-02007-0 (PMC7446187; doi:10.1186/s12882-020-02007-0)
Supplement: Supplementary file 2 — Additional file 2: Fig. S2. Immunofluorescence images of the first renal biopsy at age 5. Immunofluorescence images of renal biopsy at age 5. Immunoglobulins (IgG, IgM, and IgA) and complement proteins (C3 and C1q) were diffusely deposited along the capillary wall and expressed at similar levels. [file 12882_2020_2007_MOESM2_ESM.pdf]

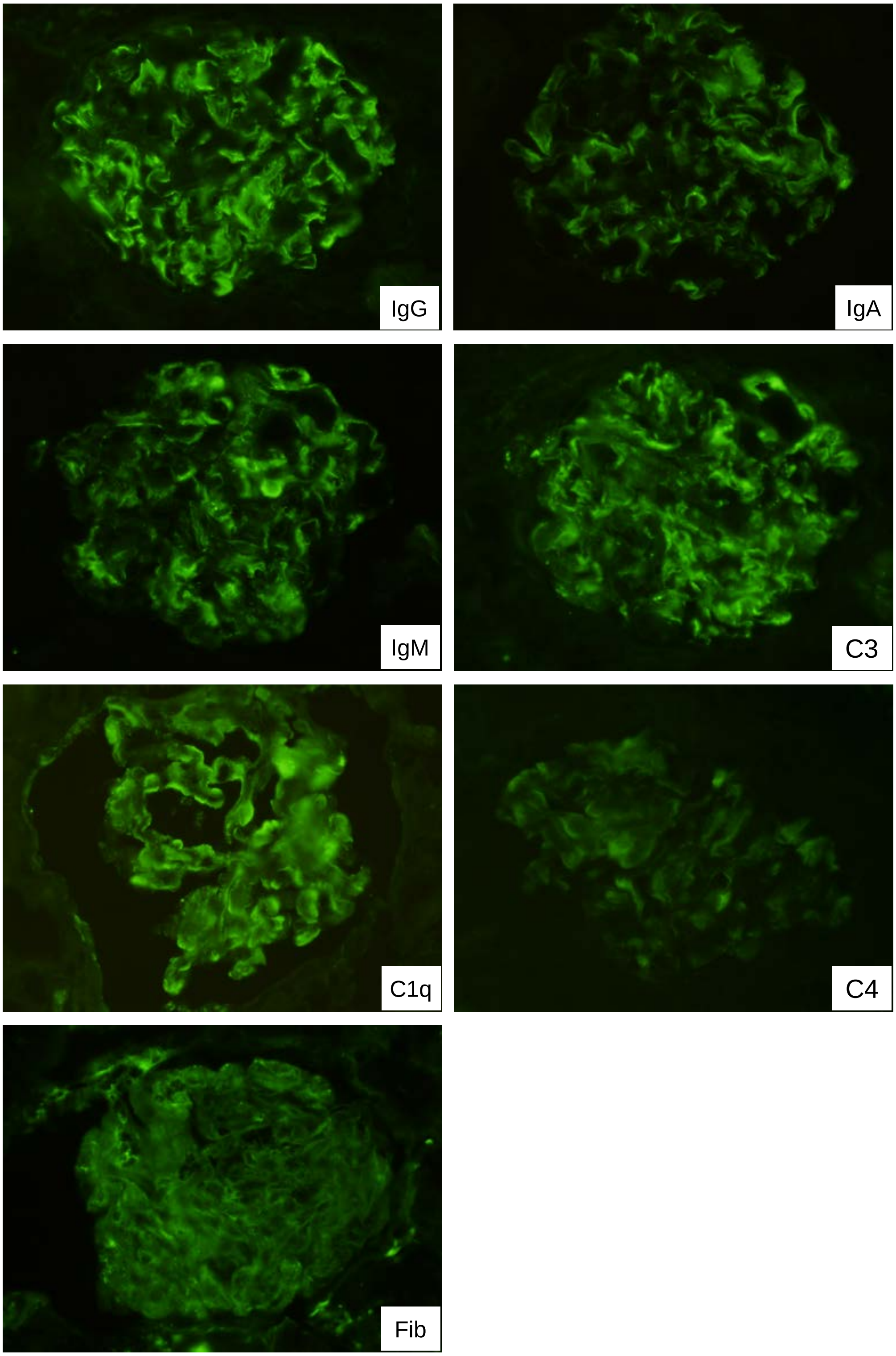

Figure S2. Immunofluorescence images of the first renal biopsy at age 5

Immunofluorescence images of renal biopsy at age 5. Immunoglobulins (IgG, IgM, and IgA) and complement proteins (C3 and C1q) were diffusely deposited along the capillary wall and expressed at similar levels.
