# Supplementary material for: Immune-complex glomerulonephritis with a membranoproliferative pattern in Frasier syndrome: a case report and review of the literature
Source: BMC Nephrol. 2020 Aug 24;21:362. doi: 10.1186/s12882-020-02007-0 (PMC7446187; doi:10.1186/s12882-020-02007-0)
Supplement: Supplementary file 3 — Additional file 3: Fig. S3. Electron micrographs of the first biopsy at age 5. (a) Representative images of electron-dense deposits in the paramesangial area (asterisks). Thickness and contour of the GBM appear generally normal, while there was partial scalloping in the paramesangial region. Scale bar, 2 μm. (b) Enlarged view of the boxed area in (a). Some portion of the GBM was slightly thickened showing double layers of dense matrix (arrowheads). Scale bar, 1 μm. (c) Electron-dense deposits in the subendothelial and paramesangial spaces (asterisks), with occasional thickening of the adjacent GBM. In podocytes, there were numerous cytoplasmic vacuoles and deformities, including foot-process effacement and microvilli formation. Scale bar, 2 μm. (d) Enlarged view of the boxed area in (c). The GBM appeared abnormally thickened with granular, subendothelial, electron-dense deposits (asterisks). Scale bar, 1 μm. [file 12882_2020_2007_MOESM3_ESM.pdf]

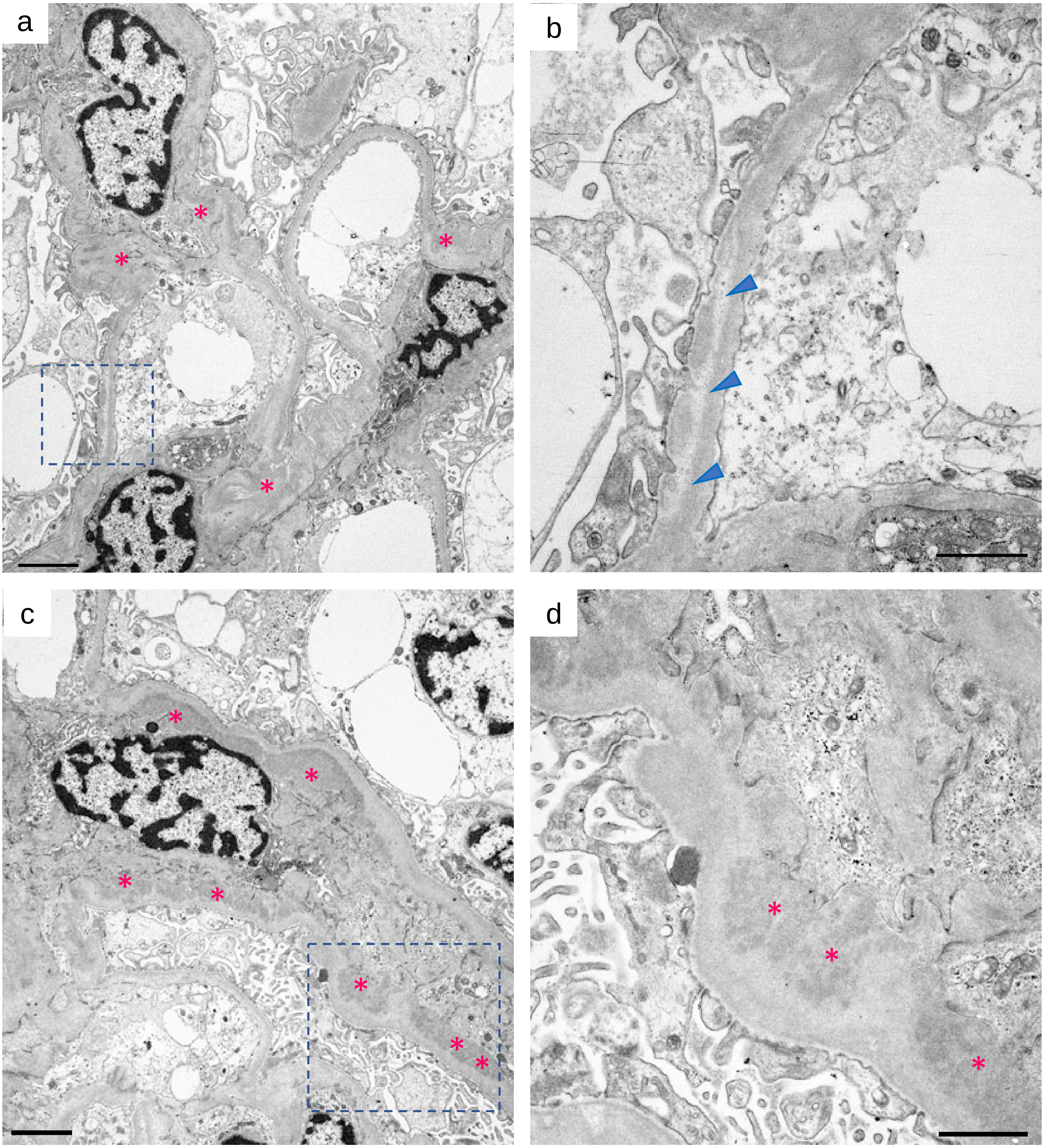

Figure S3. Electron micrographs of the first biopsy at age 5

(a) Representative images of electron-dense deposits in the paramesangial area (asterisks). Thickness and contour of the GBM appear generally normal, while there was partial scalloping in the paramesangial region. Scale bar, 2 µm. (b) Enlarged view of the boxed area in (a). Some portion of the GBM was slightly thickened showing double layers of dense matrix (arrowheads). Scale bar, 1 µm. (c) Electron-dense deposits in the subendothelial and paramesangial spaces (asterisks), with occasional thickening of the adjacent GBM. In podocytes, there were numerous cytoplasmic vacuoles and deformities, including foot-process effacement and microvilli formation. Scale bar, 2 µm. (d) Enlarged view of the boxed area in (c). The GBM appeared abnormally thickened with granular, subendothelial, electron-dense deposits (asterisks). Scale bar, 1 µm.
