# Supplementary material for: Immune-complex glomerulonephritis with a membranoproliferative pattern in Frasier syndrome: a case report and review of the literature
Source: BMC Nephrol. 2020 Aug 24;21:362. doi: 10.1186/s12882-020-02007-0 (PMC7446187; doi:10.1186/s12882-020-02007-0)
Supplement: Supplementary file 4 — Additional file 4: Table S1. Summary of serial immunofluorescence studies. [file 12882_2020_2007_MOESM4_ESM.docx]

**Table S1. Summary of serial immunofluorescence studies**

|  | First biopsy | |  | Second biopsy | |  | Third biopsy | |
| --- | --- | --- | --- | --- | --- | --- | --- | --- |
|  | Intensity | Pattern |  | Intensity | Pattern |  | Intensity | Pattern |
| IgG | 1+ | Diffuse MC |  | 1+ | Diffuse, capillary fringe |  | +/- | Partial, capillary |
| IgA | 1+ | Diffuse MC |  | +/- | Diffuse, capillary |  | - |  |
| IgM | 1+ | Diffuse MC |  | 1+ | Diffuse, capillary fringe |  | 2+ | Diffuse, capillary fringe |
| C3 | 1+ | Diffuse MC |  | +/- | Diffuse, capillary |  | 1+ | Partial, capillary |
| C1q | 1+ | Diffuse MC |  | - |  |  | +/- | Partial, capillary fringe |
| C4 | +/- | Diffuse MC |  | - |  |  | +/- | Partial, capillary fringe |
| Fib | - |  |  | - |  |  | + | Partial, capillary |

MC: Mesangiocapillary.
