# Supplementary material for: Immune-complex glomerulonephritis with a membranoproliferative pattern in Frasier syndrome: a case report and review of the literature
Source: BMC Nephrol. 2020 Aug 24;21:362. doi: 10.1186/s12882-020-02007-0 (PMC7446187; doi:10.1186/s12882-020-02007-0)
Supplement: Supplementary file 5 — Additional file 5: Fig. S4. Immunofluorescence images of the second renal biopsy at age 6. Representative immunofluorescence images of the second renal biopsy at age 6. Dense IgM deposition (1+) localized in the mesangial area, as well as in the periphery of glomerular capillaries, forming a fringe-like pattern (arrowheads). IgG co-deposited (1+) to a lesser degree than IgA, C3(+/−). Overall, immunocomplex deposition was significantly lower than that found in the first biopsy, indicating successful removal of ICs by immunosuppressive therapy. [file 12882_2020_2007_MOESM5_ESM.pdf]

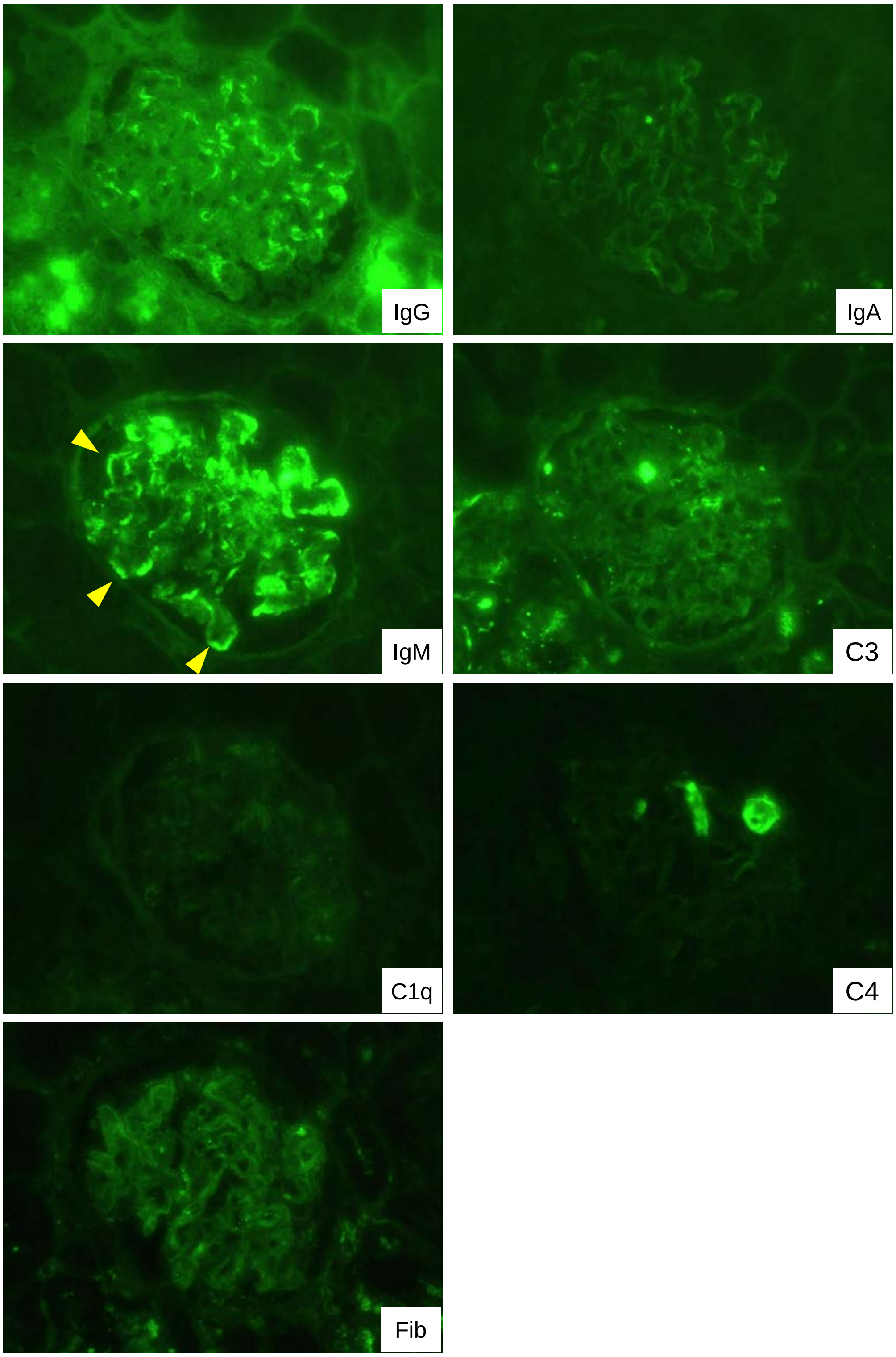

Figure S4. Immunofluorescence images of the second renal biopsy at age 6

Representative immunofluorescence images of the second renal biopsy at age 6. Dense IgM deposition (1+) localized in the mesangial area, as well as in the periphery of glomerular capillaries, forming a fringe-like pattern (arrowheads). IgG co-deposited (1+) to a lesser degree than IgA, C3(+/−). Overall, immunocomplex deposition was significantly lower than that found in the first biopsy, indicating successful removal of ICs by immunosuppressive therapy.
