# Supplementary material for: Immune-complex glomerulonephritis with a membranoproliferative pattern in Frasier syndrome: a case report and review of the literature
Source: BMC Nephrol. 2020 Aug 24;21:362. doi: 10.1186/s12882-020-02007-0 (PMC7446187; doi:10.1186/s12882-020-02007-0)
Supplement: Supplementary file 6 — Additional file 6: Fig. S5. Histology of the second renal biopsy at age 6. (a, b) Representative images of the second renal biopsy at age 6. Most glomeruli showed an increase in mesangial matrices, whereas some displayed segmental sclerosis (arrows). Foam cells accumulated primarily in the interstitium (arrowheads). (a) Scale bar, 250 μm. (b) Scale bar, 100 μm. (c) Increases in mesangial matrix were more pronounced in perihilar regions (arrow). Tubular atrophy and dilatation (asterisk) were observed in the interstitium adjacent to the sclerosing glomeruli. Histology resembled FSGS more closely, despite the coexistence of some MPGN characteristics. Scale bar, 50 μm. (d) Representative image of glomeruli with global mesangial proliferation and aggregation of foam cells within the capillary lumen and Bowman’s space (arrowheads). Some glomeruli exhibited tuft adhesion and segmental sclerosis (arrow). Scale bar, 50 μm; periodic acid-Schiff staining. (a–c) Periodic acid methenamine silver staining. [file 12882_2020_2007_MOESM6_ESM.pdf]

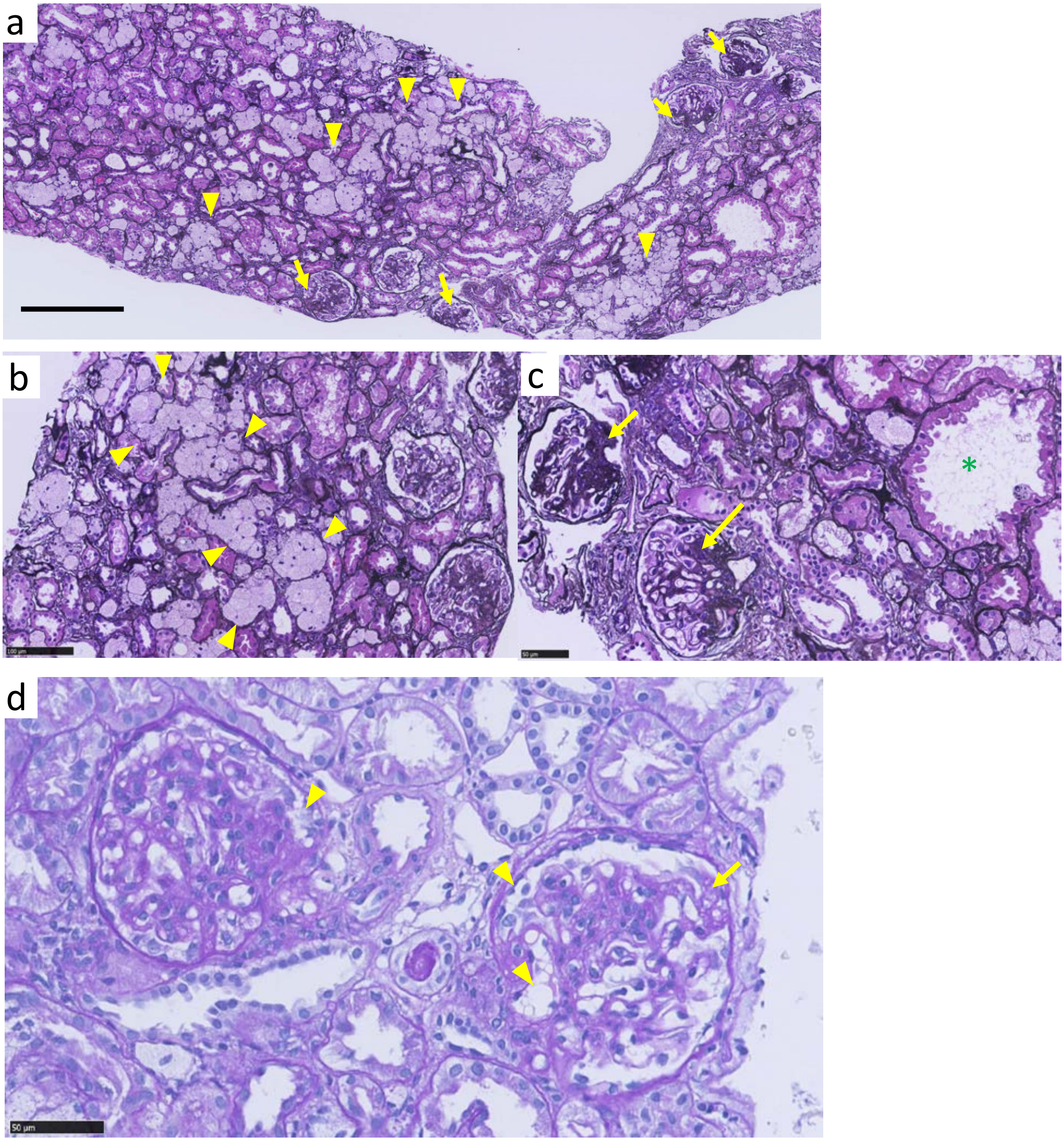

Figure S5. Renal histology of the second renal biopsy at age 6

(a, b) Representative images of the second renal biopsy at age 6. Most glomeruli showed an increase in mesangial matrices, whereas some displayed segmental sclerosis (arrows). Foam cells accumulated primarily in the interstitium (arrowheads). (a) Scale bar, 250 µm. (b) Scale bar, 100 µm. (c) Increases in mesangial matrix were more pronounced in perihilar regions (arrow). Tubular atrophy and dilatation (asterisk) were observed in the interstitium adjacent to the sclerosing glomeruli. Histology resembled FSGS more closely, despite the coexistence of some MPGN characteristics. Scale bar, 50 µm. (d) Representative image of glomeruli with global mesangial proliferation and aggregation of foam cells within the capillary lumen and Bowman's space (arrowheads). Some glomeruli exhibited tuft adhesion and segmental sclerosis (arrow). Scale bar, 50 µm; periodic acid-Schiff staining. (a–c) Periodic acid methenamine silver staining.
