# Supplementary material for: Immune-complex glomerulonephritis with a membranoproliferative pattern in Frasier syndrome: a case report and review of the literature
Source: BMC Nephrol. 2020 Aug 24;21:362. doi: 10.1186/s12882-020-02007-0 (PMC7446187; doi:10.1186/s12882-020-02007-0)
Supplement: Supplementary file 7 — Additional file 7: Fig. S6. Electron micrographs of the second biopsy at age 6. Ultrastructure of glomeruli after immunosuppressive therapy. (a) The amount of capillary deposition decreased relative to that of the first biopsy. However, some deposits remained in the subendothelial (arrow) and subepithelial regions (arrowheads). Double arrows indicate deposit-free capillary wall. Scale bar, 2 μm. (b) Scalloping and irregular thickening of the GBM observed along with electron-lucent matrix expansion (asterisks). Podocytes were deformed with cytoplasmic vacuolization, foot-process effacement, and microvilli formation. Scale bar, 2 μm. (c) Mesangial matrices and fragmented electron-dense depositions (asterisks) increased in the paramesangial regions. Scale bar, 2 μm. (d) The GBM was abnormally thickened and partially split due to an accumulation of fine granular deposits in the subendothelial and subepithelial regions, as well as mesangial interposition. Scale bar, 2 μm. [file 12882_2020_2007_MOESM7_ESM.pdf]

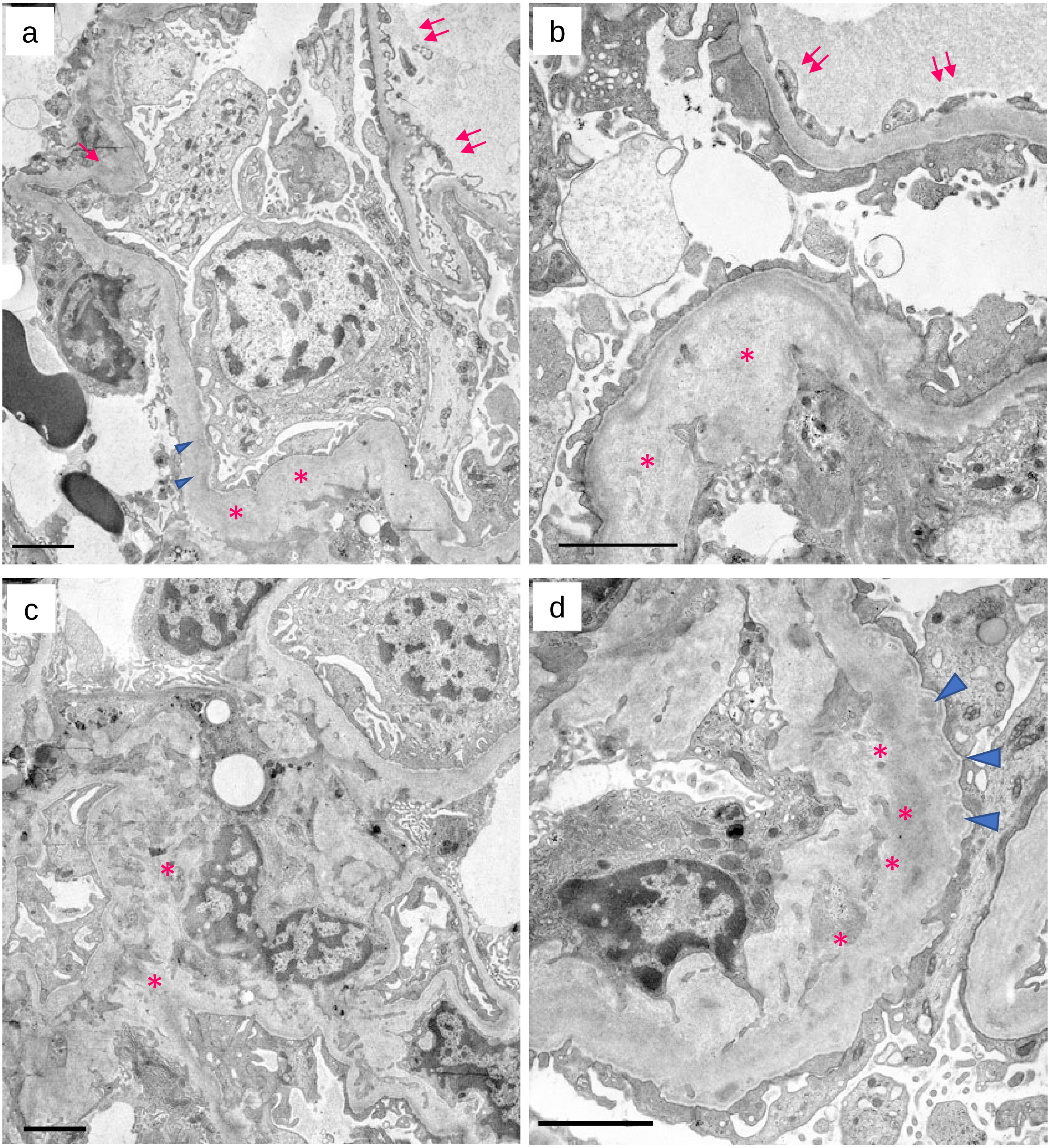

Figure S6. Electron micrographs of the second biopsy at age 6

Ultrastructure of glomeruli after immunosuppressive therapy. (a) The amount of capillary deposition decreased relative to that of the first biopsy. However, some deposits remained in the subendothelial (arrow) and subepithelial regions (arrowheads). Double arrows indicate deposit-free capillary wall. Scale bar, 2 µm. (b) Scalloping and irregular thickening of the GBM observed along with electron-lucent matrix expansion (asterisks). Podocytes were deformed with cytoplasmic vacuolization, foot-process effacement, and microvilli formation. Scale bar, 2 µm. (c) Mesangial matrices and fragmented electron-dense depositions (asterisks) increased in the paramesangial regions. Scale bar, 2 µm. (d) The GBM was abnormally thickened and partially split due to an accumulation of fine granular deposits in the subendothelial and subepithelial regions, as well as mesangial interposition. Scale bar, 2 µm.
