# Supplementary material for: Immune-complex glomerulonephritis with a membranoproliferative pattern in Frasier syndrome: a case report and review of the literature
Source: BMC Nephrol. 2020 Aug 24;21:362. doi: 10.1186/s12882-020-02007-0 (PMC7446187; doi:10.1186/s12882-020-02007-0)
Supplement: Supplementary file 8 — Additional file 8: Fig. S7. Immunofluorescence images of the third renal biopsy at age 8. Dense and diffuse IgM deposition observed predominantly along the glomerular capillary. Complement proteins (C3, C1q, and C4) co-deposited with IgM in the tufts, suggesting non-specific entrapping of macromolecules due to a gradual loss of glomerular structural integrity. [file 12882_2020_2007_MOESM8_ESM.pdf]

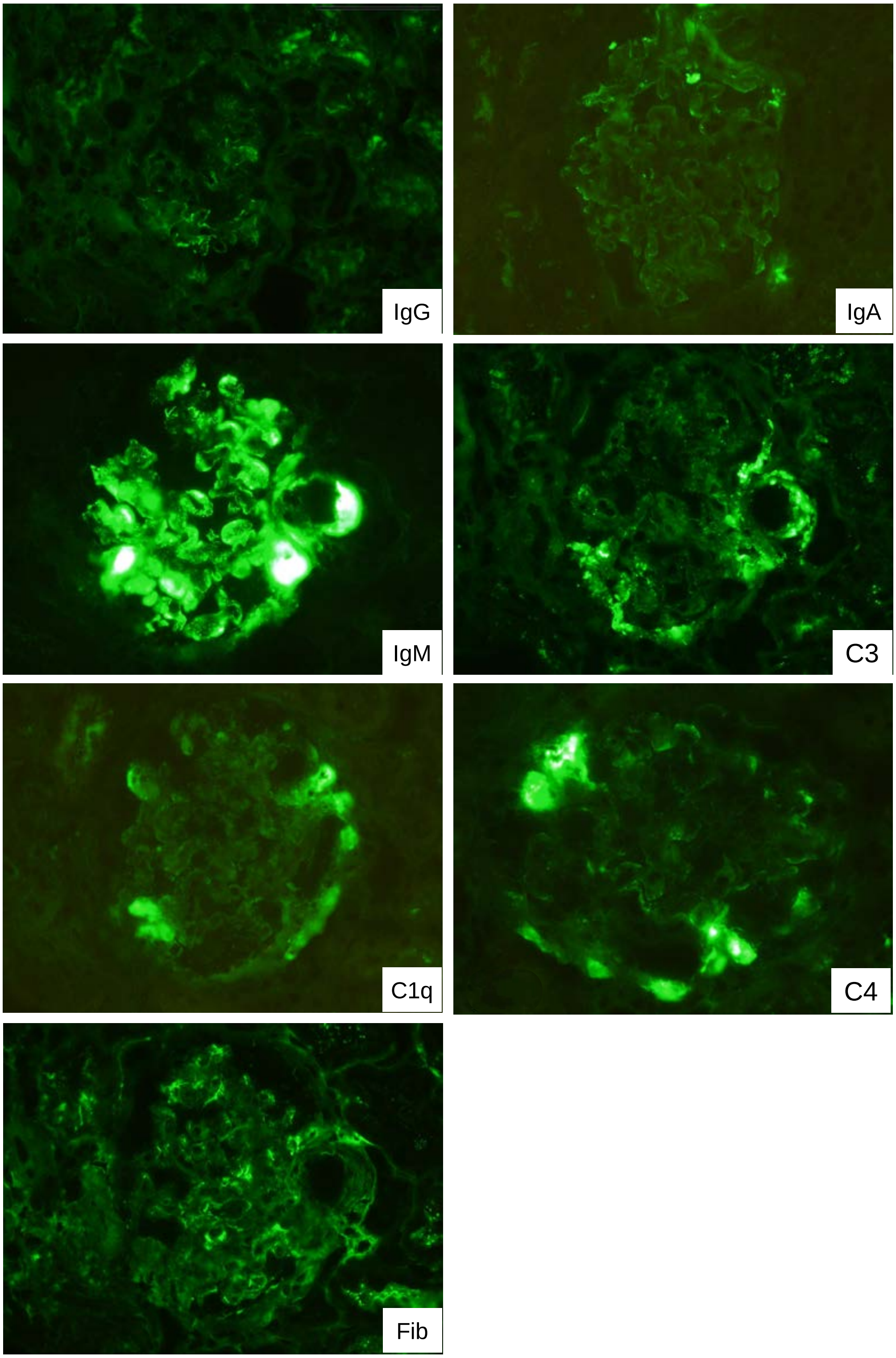

Figure S7. Immunofluorescence images of the third renal biopsy at age 8

Dense and diffuse IgM deposition observed predominantly along the glomerular capillary. Complement proteins (C3, C1q, and C4) co-deposited with IgM in the tufts, suggesting non-specific entrapping of macromolecules due to a gradual loss of glomerular structural integrity.
