# Supplementary material for: Immune-complex glomerulonephritis with a membranoproliferative pattern in Frasier syndrome: a case report and review of the literature
Source: BMC Nephrol. 2020 Aug 24;21:362. doi: 10.1186/s12882-020-02007-0 (PMC7446187; doi:10.1186/s12882-020-02007-0)
Supplement: Supplementary file 9 — Additional file 9: Fig. S8. Renal histology of the third biopsy at age 8. Representative images of the third renal biopsy at age 8. An increasing fraction of glomeruli showed segmental-to-global sclerosis (arrows) and expansion of interstitial fibrosis (asterisks), suggesting FSGS progression Foam cells focally aggregated in the interstitium (arrowheads). However, overall cell density was significantly lower than in previous biopsies. (a) Scale bar, 250 μm. (b) Scale bar, 100 μm. (c) Arrows indicate the double-contour in the glomerular capillary, and the arrowhead indicates segmental luminal dilation and foam cell accumulation. Scale bar, 50 μm. (d, e) Glomerulus with increased mesangial matrix and tuft adhesion (arrow) along with foam cell accumulation in Bowman’s space and the capillary lumen (arrowheads). (e) Double arrowheads indicate hyaline nodules in the vascular pole. Scale bar, 50 μm; The arteriole (asterisk) appeared normal. (a–c) Periodic acid methenamine silver staining, (d, e) periodic acid-Schiff staining. [file 12882_2020_2007_MOESM9_ESM.pdf]

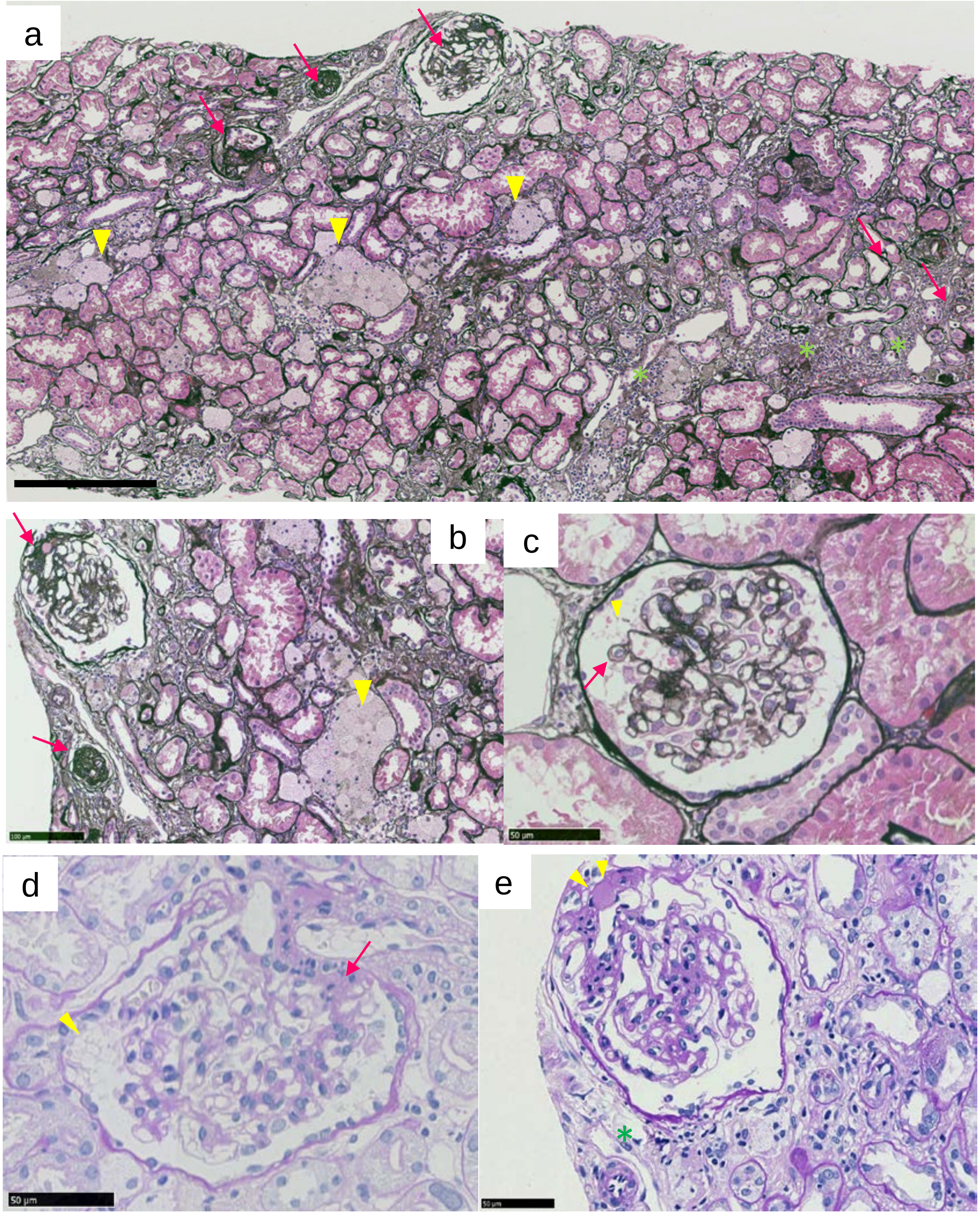

Figure S8. Renal histology of the third biopsy at age 8

Representative images of the third renal biopsy at age 8. An increasing fraction of glomeruli showed segmental-to-global sclerosis (arrows) and expansion of interstitial fibrosis (asterisks), suggesting FSGS progression Foam cells focally aggregated in the interstitium (arrowheads). However, overall cell density was significantly lower than in previous biopsies. (a) Scale bar, 250 µm. (b) Scale bar, 100 µm. (c) Arrows indicate the double-contour in the glomerular capillary, and the arrowhead indicates segmental luminal dilation and foam cell accumulation. Scale bar, 50 µm. (d, e) Glomerulus with increased mesangial matrix and tuft adhesion (arrow) along with foam cell accumulation in Bowman's space and the capillary lumen (arrowheads). (e) Double arrowheads indicate hyaline nodules in the vascular pole. Scale bar, 50 µm; The arteriole (asterisk) appeared normal. (a–c) Periodic acid methenamine silver staining, (d, e) periodic acid-Schiff staining.
