# Supplementary material for: Immune-complex glomerulonephritis with a membranoproliferative pattern in Frasier syndrome: a case report and review of the literature
Source: BMC Nephrol. 2020 Aug 24;21:362. doi: 10.1186/s12882-020-02007-0 (PMC7446187; doi:10.1186/s12882-020-02007-0)
Supplement: Supplementary file 10 — Additional file 10: Table S2. A list of the genes included in the next-generation sequencing panel screening. [file 12882_2020_2007_MOESM10_ESM.pdf]

Table S2. A list of the genes included in the next-generation sequencing panel screening.

ACE	*CLCNKB*	*GLEPP1(PTPRO)*	*NUP107*	*TRPC6*
ACTN4	*CLDN16*	*HNF1B*	*NUP205*	*TRPM6*
ADCK4	*CLDN19*	*INF2*	*NUP93*	*TTC21B*
AGT	*CNNM2*	*ITGA3*	*OCRL*	*WDR73*
AGTR1	*COL4A3*	*ITGB4*	*PAX2*	*WNK1*
ANLN	*COL4A4*	*KANK1*	*PDSS2*	*WNK4*
ARHGAP24	*COL4A5*	*KANK2*	*PLCE1(NPHS3)*	*WT1*
ARHGDIA	*COQ2*	*KANK4*	*PTPRO*	*XPO5*
ATP6V0A4	*COQ6*	*KCNA1*	*REN*	
ATP6V1B1	*CRB2*	*KCNJ1*	*SCARB2*	
BSND	*CTNS*	*KCNJ10*	*SCNN1A*	
C3	*CUBN*	*KLHL3*	*SCNN1B*	
CASR	*CUL3*	*LAMB2*	*SCNN1G*	
CD2AP	*DGKE*	*LMX1B*	*SLC12A1*	
CFB	*EFG*	*MCP (CD46)*	*SLC12A3*	
CFH	*EMP2*	*MYH9*	*SLC26A3*	
CFI	*FAT1*	*MYO1E*	*SLC4A1*	
CFTR	*FN1*	*NPHS1*	*SLC4A4*	
CLCN5	*FXYD2*	*NPHS2*	*SMARCAL1*	
CLCNKA	*GLA*	*NR3C2*	*THBD*	
